# Supplementary material for: Play like me: Similarity in playfulness promotes social play
Source: PLoS One. 2019 Oct 24;14(10):e0224282. doi: 10.1371/journal.pone.0224282 (PMC6812795; doi:10.1371/journal.pone.0224282)
Supplement: S1 Table — (DOCX) [file pone.0224282.s003.docx]

**S1 Table. Description of the main phases of the experiment.**

| **Phase** | **Procedure** | **Measurement** | **Outcome measures** |
| --- | --- | --- | --- |
| **Arrival** | Sorting into 19 cages of 4 non-littermates | None | None |
| **Play-in-Pairs Test 1 (PIP 1)** | Three play sessions per rat, each with a different cage mate | Individual playfulness levels and classification of rats into playfulness categories: H = high, I = intermediate, L = low | Pinning and Attack to Nape |
| **Resorting** | Resorting into 25 cages of 3 unfamiliar non-littermates;  5 treatment groups (HHH, HII, III, LII, LLL) based on playfulness classification in PIP 1 | None | None |
| **Three Home Cage Observations  (Weeks 8, 10, 12 of age)** | Continuous observation of play behaviour in the home cage during the second quarter of each of the first four hours of the dark phase | Social play behaviour | Pinning and Attack to Nape |
| **Play-in-Pairs Test 2 (PIP 2)** | Two play sessions per rat, each with a different cage mate | Consistency of playfulness between PIP 1 and PIP 2, and comparison with home cage play levels | Pinning and Attack to Nape |
| **Social Preference Test** | I rats of the heterogeneous treatment groups were given a social preference test | Preference for dissimilar (H or L) or similar (I) cage mate | Time spent in proximity of either the dissimilar or the similar cage mate |
