# Supplementary material for: Play like me: Similarity in playfulness promotes social play
Source: PLoS One. 2019 Oct 24;14(10):e0224282. doi: 10.1371/journal.pone.0224282 (PMC6812795; doi:10.1371/journal.pone.0224282)
Supplement: S2 Table — (DOCX) [file pone.0224282.s004.docx]

**S2 Table. Classification of rats into High (H; n=20), Intermediate (I; n=35) and Low (L; n=20) playfulness categories, and assignment to treatment groups and cages.**

| **Rank** | **Subject** | **Counts of attacks to nape + pinnings** | **Playfulness Category** | **Treatment Group** | **Assigned Cage** |
| --- | --- | --- | --- | --- | --- |
| 1 | D4 | 122 | H | HII | 1 |
| 2 | B2 | 121 | H | HII | 2 |
| 3 | K2 | 120 | H | HII | 3 |
| 4 | R1 | 117 | H | HII | 4 |
| 5 | L4 | 112 | H | HII | 5 |
| 6 | K1 | 107 | H | HHH | 11 |
| 8 | L1 | 102 | H | HHH | 11 |
| 8 | D1 | 102 | H | HHH | 15 |
| 8 | G4 | 102 | H | HHH | 14 |
| 10 | H4 | 99 | H | HHH | 13 |
| 11.5 | F2 | 96 | H | HHH | 11 |
| 11.5 | G2 | 96 | H | HHH | 12 |
| 13.5 | C3 | 94 | H | HHH | 14 |
| 13.5 | G3 | 94 | H | HHH | 13 |
| 15 | N3 | 93 | H | HHH | 15 |
| 16.5 | A4 | 90 | H | HHH | 15 |
| 16.5 | F4 | 90 | H | HHH | 14 |
| 18 | S3 | 89 | H | HHH | 13 |
| 19.5 | S2 | 88 | H | HHH | 12 |
| 19.5 | T3 | 88 | H | HHH | 12 |
| 21 | B3 | 86 | I | III | 23 |
| 22 | U2 | 85 | I | III | 25 |
| 23 | R3 | 84 | I | III | 25 |
| 24.5 | E2 | 83 | I | III | 23 |
| 24.5 | P4 | 83 | I | III | 25 |
| 26 | C1 | 82 | I | III | 23 |
| 27 | Q2 | 78 | I | III | 24 |
| 28 | U1 | 75 | I | III | 24 |
| 29.5 | A2 | 73 | I | III | 24 |
| 29.5 | L3 | 73 | I | LII | 7 |
| 31 | K3 | 72 | I | HII | 1 |
| 32.5 | N4 | 71 | I | LII | 7 |
| 32.5 | S4 | 71 | I | LII | 6 |
| 34.5 | E1 | 70 | I | LII | 8 |
| 34.5 | S1 | 70 | I | HII | 2 |
| 36.5 | D3 | 69 | I | LII | 8 |
| 36.5 | P1 | 69 | I | LII | 6 |
| 38 | F3 | 68 | I | HII | 1 |
| 39.5 | D2 | 67 | I | HII | 3 |
| 39.5 | E3 | 67 | I | LII | 9 |
| 41.5 | H2 | 64 | I | HII | 3 |
| 41.5 | W1 | 64 | I | HII | 2 |
| 43 | N2 | 62 | I | HII | 4 |
| 44 | T2 | 61 | I | LII | 9 |
| 45.5 | K4 | 60 | I | HII | 5 |
| 45.5 | L2 | 60 | I | LII | 10 |
| 47 | W2 | 58 | I | HII | 4 |
| 49.5 | M3 | 56 | I | LII | 10 |
| 49.5 | C2 | 56 | I | HII | 5 |
| 49.5 | G1 | 56 | I | III | 21 |
| 49.5 | M2 | 56 | I | III | 22 |
| 52 | H1 | 55 | I | III | 22 |
| 54.5 | T1 | 53 | I | III | 22 |
| 54.5 | H3 | 53 | I | III | 21 |
| 54.5 | A3 | 53 | I | III | 21 |
| 54.5 | Q4 | 53 | L | LLL | 16 |
| 57.5 | Q3 | 52 | L | LLL | 17 |
| 57.5 | T4 | 52 | L | LLL | 18 |
| 59 | E4 | 50 | L | LLL | 16 |
| 60.5 | B4 | 49 | L | LLL | 19 |
| 60.5 | N1 | 49 | L | LLL | 17 |
| 63 | P3 | 48 | L | LLL | 17 |
| 63 | C4 | 48 | L | LLL | 16 |
| 63 | U4 | 48 | L | LLL | 18 |
| 65 | A1 | 45 | L | LLL | 20 |
| 67 | R2 | 44 | L | LLL | 20 |
| 67 | P2 | 44 | L | LLL | 19 |
| 67 | W3 | 44 | L | LLL | 18 |
| 69 | M4 | 43 | L | LLL | 19 |
| 70.5 | Q1 | 41 | L | LLL | 20 |
| 70.5 | R4 | 41 | L | LII | 6 |
| 72.5 | F1 | 40 | L | LII | 7 |
| 72.5 | M1 | 40 | L | LII | 8 |
| 74 | W4 | 36 | L | LII | 9 |
| 75 | U3 | 28 | L | LII | 10 |

Note: Letters in the Subject column (A to W) indicate the 19 cages of 4 rats before the resorting procedure.
Means ± SD of treatment groups: HHH 95.4 ± 6.0, HII 82.2 ± 27.0, III 70.4 ± 14.0, LII 56.8 ± 15.4, LLL 47.4 ± 3.6.
