# Supplementary material for: Play like me: Similarity in playfulness promotes social play
Source: PLoS One. 2019 Oct 24;14(10):e0224282. doi: 10.1371/journal.pone.0224282 (PMC6812795; doi:10.1371/journal.pone.0224282)
Supplement: Attachment — Submitted filename: Responses to Reviewers.docx [file pone.0224282.s005.docx]

**Cover letter - Article submission Lampe et al.**

Badestrasse 13, 48149 - Münster, Germany

Dr. Luca Melotti

Department of Behavioural Biology

University of Münster

Tel.: +49 25183 21006
melotti@uni-muenster.de

Münster, 31^st^ of July, 2019

Dear Editor

We hereby wish to submit our manuscript entitled “Play like me: similarity in playfulness promotes social play” for publication as a research article in *PLOS ONE*. This manuscript has previously been submitted to *PLOS ONE* (reference to original submission: PONE-D-17-25569) and reviewed by Prof. Elisabetta Palagi (Editor) and three anonymous Reviewers, but the review process was stopped because we did not resubmit the revised manuscript within the required six-month time period.
We have now carefully revised the manuscript according to the Reviewers´ comments and believe that the manuscript has improved considerably. If possible, we would kindly ask that this manuscript is reviewed again by the original Editor and Reviewers since we have specifically addressed their comments. To this end, we are submitting our Response to Reviewers file (see below). We would also be happy to submit a copy of the manuscript with track changes.

This research article provides valuable new insight into the understanding of social play development in relation to individual playfulness and highlights the importance of social group composition and compatibility between individuals for the outcome of experiments and for animal welfare.

We hope that you will find our manuscript suitable for publication and look forward to hearing from you.

Thank you for your consideration and do not hesitate to contact me for further information.

Best wishes

Luca Melotti

**Response to Reviewers**

We would like to thank the Reviewers for their very helpful and constructive comments, which we think led to a substantial improvement of our manuscript. Please find our responses below each comment.

**Reviewer #1:**

The present MS deals with the issue of "play contagion" in male rats during adolescence. The authors investigated if differences in personality traits (playfulness propensity) may affect the development of social play among group members. Even if the issue of play and emotional contagion is very interesting, there are many theoretical and methodological flaws that need to be addressed before the complete acceptance of the MS. I report my concern chronologically below.

INTRODUCTION
lines 49-52: the authors pass abruptly from the issue of Rough-&-Tumble play to that of animal welfare, but what is the linkage between the two?

We made changes at Lines 51-54 highlighting how rough-and-tumble play is linked to positive affective states, which we think clarifies the transition to animal play being relevant for animal welfare.

lines 58-59: some studies on primates demonstrated that play behaviour may occur also when animals face tension and competitive condition such as pre-feeding time. Please, address this issue.

We have reformulated this paragraph and added relevant references (Lines 61-66) to explain that the association between play and animal welfare is context-dependent.

lines 59-61: really, I do not understand the linkage between the absence of play during fitness threats and the association of this behaviour with positive emotions. See also my previous comment. Please, clarify and add citations.

We rephrased the concluding sentence of this paragraph (Lines 65-68) to clarify that on one hand, the association between play and animal welfare is context-dependent, and on the other hand the literature referred to in the whole paragraph points towards converging evidence that play is associated with the experience of positively valenced emotional states.

lines 62-69: a deeper definition of the fundamental concepts (empathy, emotional contagion, rapid mimicry and positive emotion) and an explanation of the linkage between them are needed.

We added a paragraph with these definitions at Lines 69-81.

line 71: which negative emotions? Please specify.

We specified which negative emotion each cited article referred to (Lines 88-89).

lines 72-78: the authors report only studies on emotional contagion in humans, but in the last years some studies have demonstrated also emotional contagion in animals (e.g. primates, canids). Please, deal with this issue.

We have now referred to recent studies on positive emotional contagion in primates and canids at Lines 90-93.

line 79: change the sentence as follows "In rats, few studies..."

Changed, thank you.

lines 91-94: please, define better "short-term difference" and "long-term welfare implications". Moreover, emotional contagion and motivation are two different concepts: the linkage between the two is not clear in the text.

We clarified and expanded this part (Lines 104-113) to better explain the limitations of the previous findings on emotional contagion through play behaviour in rats. “Short-term” refers now more specifically to the social isolation treatments used in previous studies to alter play motivation before assessing contagion. The “long-term” approach of our study is now more clearly defined (from adolescence to early adulthood).


lines 95-96: the authors stated that play may assess and promote animal welfare only if its contagious traits are expressed consistently along the lifetime of animals. Nevertheless, in the majority of species play follows an U pathway by increasing from infancy to juvenility and by "physiologically" decreasing towards adulthood. Other affinitive interactions put into play. Please, deal with this issue and add citations.

Following the clarification of the life phases (adolescence to early adulthood) to which we refer in our study at Lines 110-112, we now explicitly state that the contagious properties of play should be present for at least part of a lifetime in order to substantially improve animal welfare (Lines 113-115). We would consider the period from adolescence to early adulthood, when play levels in rats are still relatively high, as a substantial part of a lifetime.

line 97: please, define "personality trait playfulness".

We added this definition at Lines 116-118.


lines 107-108: generally a social group is composed by individuals that show different personality combinations. Please, explain better the peculiarity of your approach.

We clarified that “This approach allows for the systematic exploration of play development over time based on known initial differences in playfulness between group members” at Lines 125-127.

lines 109-115: move this part into the Method section.

We partially rephrased this part but we think it is necessary to keep it where it is to help the reader understand the formulation of the hypothesis in the last paragraph of the Introduction.


lines 116-126: I suggest to re-formulate your Predictions with more clear theoretical framework and definition. For example, why do you expect that heterogeneous groups play less than HHH group and more that LLL one?

We have realised that Hypothesis (iv) was not formulated clearly and lacked support from the literature, so we have now rephrased our expectation that “one or more homogenous groups would play more than the heterogeneous groups, as cage mates with similar motivation to play have been found to avoid each other less (Douglas et al., 2004; Varlinskaya et al., 1999)” at Lines 145-147.


METHODS
lines 138-141: did you make 19 cages composed by 4 non-littermate rats each? On what basis did you define rats as bolder or shyer? Did you make a distinction before the separation in the different 19 cages? Please, clarify.

 Reviewer 1 is correct, we formed 19 cages of four non-littermates. We have now added the number 19 to clarify this. We have now explained how we counterbalanced for boldness across cages at Lines 162-163.


line 148: if you switched off light at 9 am, you applied a reverse 12:12 light:dark cycle. Why did you employ this procedure?

As rats are nocturnal animals and are most active in the dark phase, we have now explained that “a reverse 12:12 light:dark cycle (lights off at 9 am) allowing us to carry out behavioural tests and observations during the dark, active phase for the rats” was used (Lines 170-172).

line 149: change the sentence as follows "Initial habituation to be handled by..."

Changed, thank you.

lines 150-156: really, I do not understand the meaning of this part: it refers to another study. Rather this handling procedure may positively affect the habituation process of the rats. Please, clarify.

 We see that our explanation was not detailed enough so we have clarified that “These procedures involved gradual and short (a few minutes per day) exposures to white light during the dark phase in order to take photos of the face, and were not performed on the days when the tests and observations of the present study occurred” (Lines 176-179).

line 157: why did you decide to test animals only during dark phase?

This question is now addressed at Lines 170-172.

As overall, the experimental design needs to be re-organized in order to facilitate the comprehension of the reader. The experimental procedure is composed by many phases difficult to be followed and understood both from the text and the Figure 1. Maybe a Table may help in visualizing the schedule and the structure of each phase.

 We made some rephrasing at Lines 183-198 to clarify the description of the main phases of the experiment in the text. We also added a table (S1 Table) providing a description of each experimental phase, and edited Figure 1 to display the experimental timeline more clearly.

line 161: "...according to their playfulness"

Changed, thank you.

lines 161-163: really, I do not understand this part. Did you pick up the rats from the initial 19 cages and make new groups composed by 3 rats each (25 cages in total)?

 Yes, Reviewer 1 is correct. We made some rephrasing to clarify this at Lines 185-187.


lines 168-171: which method did you employ for assessing home cage play? The all occurrences sampling method? Why did you carry out observation only at 8, 10 and 12 weeks of age and not continuously during the weeks? Why did you perform the Play-in-Pairs test (hereafter PIP) at the eleventh week and not at the end of the home cage observation?

 We have now clarified that the section this comment refers to is just the “Experimental design overview” and referred to specific section for further details. Home cage play was assessed continuously during the second quarter of each of the first four hours of the dark phase (Lines 263-265). Home cage observations where performed at three separate age points to cover the longer time period as possible between adolescence and early adulthood (1h per animal per age point, for a total of 225 h of focal observation; Lines 265-266). At Line 261 we had explained that the second PIP allowed for comparison with home cage play levels; thus, we think that positioning the PIP test between the second and third home cage observations allowed for a better comparison between the two.

line 171: "...and to make a comparison with..."

Changed.

line 172: "...performed a social preference test for assessing their..."

Also changed.

lines 179-181: did the PIP consist in recording rat play sessions? Did you perform PIP before the division of the rats in 19 cages composed by 4 individuals each? Please, clarify.

 We have clarified this section (Lines 205-210) by explaining how many pair combinations per cage were possible and how many measures per rat were taken in order to determine playfulness. We also specified that the animals were already housed in cages of 4 animals when the PIP was conducted. In addition, the modified Figure 1 and the added S1 Table should now further clarify the order of events.


line 199: you defined pinning (PN) as a play pattern by which a rat causes the partner to rotate completely onto its back, hence the resulting play outcome after the nape (AN) is performed by "the attacker" and not by the receiver. If we consider the AN as a sort of play invitation, which does behavioural pattern represent a positive response by the receiver and thus its motivation to play?

 We have clarified the definition of PN (Lines 231-234). We also pointed out the possible limitations of not including the defence component of play in our study and provided a justification (Lines 585-593).


lines 218-219: a Table reporting the playfulness scores of the rats may be useful.

 We have now created such table (S2 Table), which is also showing to which treatment group and cage each rat was assigned.


line 230: since the play peaks were interspersed across the first four hours of dark phase, a continuous observation during this period may be more correct. Please, clarify your choice.

 We have expanded and justified our approach at Lines 266-272: “This sampling procedure was chosen based on continuous pilot observations of a subsample of cages, which revealed the presence of play peaks of relatively short duration, which were interspersed across the first four hours of the dark phase. Also, it was previously shown that a 1 h observation per day during a highly active period can allow for the collection of relatively high frequencies of play (Melotti et al., 2014). For this reason, observations focused on the first hours of the dark phase as during this period general activity levels are relatively high (Hurst et al., 1996)”.


Social Preference Test
Really, I do not understand what you measured by Social Preference Test. Did you measure only the time spent by the subject rat near the stimulus? Did you measure each social interaction (i.e. social sniffing or touching through the gates)? I am not sure about the strength of this method in measuring the social preference between the subjects. Many studies (demonstrating a strict linkage between emotional contagion and social bond) determined the social preference (and thus the quality of the relationship) by measuring the levels of interactions such as grooming, contact-sitting, social-licking, agonistic support. Please, clarify.

 We have now pointed out the limitations of the social preference test we used in the Discussion at Lines 599-606, thank you.


RESULTS
line 334: following your definition, AN and PN are performed by the same individual ("the attacker") in a sort of a sequential motor action, thus I am not surprise that the two patterns are highly correlated.

The sequential motor action AN – PN was indeed very frequent, but PN was also performed repeatedly while the cage mate was supine without AN occurring, as specified in the PN definition (Lines 231-234).


lines 337-347: did you employ Bonferroni's correction?

Yes, as described in the Data Analysis section (Lines 328-332)


Figure 3: please, add the asterisks for the significance results (at least between treatment groups).

Added.


Figure 4: please, add the asterisks for the significance results.

Added.


lines 407-415: really, I do not understand how you carried out these analyses and the Figure 5 does not help in the comprehension.

 We made minor changes to clarify that the play frequencies of the seeders where compared with the play frequencies of the pairs of I rats (Lines 448-454). We had also specified in the Data Analysis section that “The play frequencies of the pairs of I cage mates were averaged in order to have a balanced comparison between seeders and I rat pairs” (Lines 335-337).


lines 429-436: why did you make a correlation on PN frequencies? If your aim is to evaluate the distribution of this pattern during the Times at the individual level, you can simply perform a K-dependent sample test. Moreover, I am not convinced that only PN can consistently attest the play distribution over time. Please, clarify.

Correlation was used because we wanted to assess consistency, i.e. the maintenance of relative differences in play between individuals over time. As for the use of PN as the main outcome measure, we have addressed the possible limitations of our approach in the Discussion and provided relevant references, however we also referred to previous research showing that PN and Being Pinned (complete rotation) are positively correlated in Lister Hooded rats (Lines 585-593). Assuming a correlation between the main attack component (PN, positively correlated with AN) and the main defence component (Being Pinned), we expected that PN would represent playfulness personality relatively well in this strain.

DISCUSSION
I recommend to review the discussion in the light of the theoretical, methodological and statistical suggestion.

 We have done our best!

**Reviewer #2:**

The authors address a very interesting question in asking whether playfulness of one rat can have a long-lasting impact on the playfulness of cage-mates. Although the overall logic and design of the experiment is sound, the execution of the design leaves much to be desired. My primary concern is that the "play" being measured in the home cages after puberty is not the same as the play initially assessed in the 1st play-in-pairs test. Although the authors suggest that their measurements are being taken through adolescence, it is more likely that the social interactions being observed are those well into adulthood since the first home cage observations are being measured at about 60 days of age (see Spear, Neurosci Behav Behav, 2000, 24, 417 for a discussion on the timing of adolescence). While these interactions are probably playful, there is ample evidence to suggest that the play of adult males is different than the play of juvenile males (e.g., Pellis and colleagues have written extensively on this topic) and, as such, may be driven by different factors. The low (although significant) correlation (r = 0.23) between play in the 1st and the 2nd play-in-pairs test seems to support this. Dominance status, for one, could easily be affecting the social interactions between rats in their home cage.

 Based on Reviewer 2´s comment we now state throughout the manuscript that the assessment of playfulness in our study was performed from adolescence to early adulthood. This led us to address the issue of having potentially measured two different types of play in early adolescence and early adulthood. Based on the assumption that hierarchy formation could affect the symmetry of playful interactions, we calculated a “Play Role” index indicating the extent to which active pins by the focal rat were reciprocated by the partner rat, with low scores indicating high reciprocation and presumably low interference from hierarchy formation (i.e., the higher the play asymmetry the stronger/more established the hierarchy would be). We found that both home cage play and, in particular, play in pairs following a short social isolation showed relatively high play symmetry (indexes of 0.37 and 0.25, respectively), more similar to what would be expected during adolescence than during adulthood. Surprisingly, the Play Role index for play in pairs did not change between the juvenile and early adulthood, suggesting that this test may induce adults to play more like juveniles. Instead, home cage play became increasingly asymmetrical with age thus it remains to be investigated whether such change in play quality may reflect a less “enjoyable” experience by at least some animals. However, our main finding that homogenous groups played more than heterogenous ones referred to the adolescence period, when play in the home cage still showed a relatively high level of play reciprocity, suggestive of a play environment still largely unaffected by hierarchy formation. We have added information on the Play Role index in the Methods (Lines 234-241 and 349-357), Results (Lines 486-492) and Discussion (Lines 633-654) sections.


I was also wondering why only pinning was analyzed even though attacks to the nape were also quantified. This is especially troubling given that the extent to which a male rat will allow itself to be fully pinned differs systematically before and after puberty (e.g., Pellis & Pellis, Dev. Psychobiololgy, 1990, 23, 215-231). Dominance status can also affect this measure as well. With these factors in mind, simply quantifying the number of times an animal is pinned makes it very difficult to interpret what differences in number of pins reflect.

We rephrased the definition of PN to clarify that it was a measure of the active component of social play (Lines 231-234). AN and PN were correlated thus only PN (the most frequent) was used for further analysis (Lines 379-381). We have also stated the possible limitations of our approach in the Discussion and provided relevant references, however we also referred to previous research showing that PN and Being Pinned (complete rotation) are positively correlated in Lister Hooded rats (Lines 585-593). Assuming a correlation between the main attack component (PN) and the main defence component (Being Pinned), we expected that PN would represent playfulness personality relatively well in this strain.

**Reviewer #3:**

The paper seeks to resolve several issues: Do rats show a consistent difference in level of playfulness, do rats show a contagion effect, do rats of differing playfulness react differently to more or less playful partners, and do rats have a preference for partners of similar playfulness? These issues are extremely important for both the experimental use of rats to evaluate neuropharmacological treatments on play and for how to interpret fluctuating levels of play with regard to making judgments on animal welfare. I have no major concerns with the experimental design, although below I note the various places where the paper would benefit from more detailed information on different aspects of that methodology. Moreover, I note that the failure in showing an effect based on the social preference test may be due to the test used and not to an actual absence of a preference. This latter point should be added as a line of consideration in the Discussion.

 Thank you for the overall positive feedback. We have added this latter point to the Discussion at Lines 599-606.


The one qualm I have with the study is in the behavioral measures of play chosen. The score(s) used essentially only reflect the initiation of play (attack) and so it remains unknown whether more playful rats are also more likely to respond to such an attack (i.e., defend) and use tactics of defense that are more likely to facilitate prolonged playful contact (see descriptive details in Pellis et al., 1997, which is cited in the paper). Thus, there are limits to using pinning as a measure of overall playfulness (see reference to Himmler et al., 2016, below). The authors have two options in dealing with this issue. First, given that they have videotaped the play sessions, they could rescore the records and obtain measures of both attack and defense and so determine whether rats of differing playfulness are consistently so across these different components (this need not be the case: for an example, see Reinhart, C. J., McIntyre, D. C., & Pellis, S. M. (2004). The development of play fighting in kindling-prone (FAST) and kindling–resistant (SLOW) rats: How does the retention of phenotypic juvenility affect the complexity of play? Developmental Psychobiology, 45: 83-92). Second, attenuate the strength of the conclusion on the strength of a “play personality” in the Discussion and reflect on what further needs to be known. If the latter is the choice made, I would recommend a follow-up paper that provides the missing information on a fuller range of playful actions taken by rats that may reflect personality differences. The authors have begun to deal with an important and largely neglected aspect of play, and so developing the methodology and the conceptual framework is important for the field.

 Thank you for the useful and constructive criticism. We have addressed these issues in different parts of the manuscript, please refer to our answers to the specific comments below.


Below are some specific concerns and suggestions for more fully articulating the methods used.
Page 5, lines 113-114: Why were HLL and HHL groups not included? I am guessing that it is because the I rats were used as a means of testing for both increases and decreases in play depending on the playfulness of the heterogeneous partner, but the rationale should be stated explicitly.

 Reviewer 3 is correct, we have now explicitly stated this at Lines 133-135.


Page 7, lines 160-161: As you did not know the play personality of the individuals prior to the Play-in-Pairs test, how could you be sure that an H and a L were not paired, yielding intermediate levels of play, leading to both of these rats being relegated as I? More detail needs to be provided as to the numerical cut-offs that were used to make the H, I, L designation. Also, the criteria used to counteract inadvertent contagion effects (as noted above) should also be detailed. Later, in lines 194-196, it is noted that 6 randomized pairings per cage will be used. Again, I am assuming that an individual’s consistency over the randomized trials will be used to decide its level of playfulness. It is unlikely that each individual will score exactly the same value in each trial, so what constitutes consistency? Again, the number ranges used to decide whether a rat is consistently an H, an L or I need to be provided. This is potentially a very useful method that could be widely adopted. For example, rats of differing playfulness may respond very differently to drug treatments. Therefore, sufficient detail need to provided here for other researchers to replicate the methodology. This is not the case at present.

 We have now clarified that PIP “consisted of repeated play sessions using all possible pair combinations of cage mates available (i.e., six pair combinations per cage, as the cages were comprised of four rats), which resulted in three play sessions per rat that were used to determine individual playfulness. This procedure provides a measure of individual playfulness that is at least partly independent from the playfulness of the play partners (Pellis & McKenna, 1992; Melotti et al., 2014; Lampe et al., 2017)” at Lines 205-210. Also, we have created S2 Table which shows the playfulness scores of each rat and to which treatment group and cage each rat was assigned.


Page 9, lines 210-212: It is not clear as to whether the PN score was given to the rat that rotated onto its back or to the rat that stood over its supine partner. If the latter, this would underestimate the playfulness of the attacker, as PN would be a subset of AN. If the former, then that would be more independent, as the PN would reflect the willingness of the supine animal to engage its partner playfully (i.e., this is a reflection of defense, as opposed to AN which is a reflection of attack). Either way, it should be noted that rats can end in the supine position of the PN by different mechanisms, with treatment and group differences in PN reflecting different patterns of play (see Himmler, S. M., Himmler, B. T., Stryjek, R., Modlińska, K., Pisula, W., & Pellis, S. M. (2016). Pinning in the play fighting of rats: A comparative perspective with some methodological recommendations. International Journal of Comparative Psychology, 29: 1-14). The actual behavior contributing to the AN+PN score for each rat greatly influences the clarity of the data and so the confidence in the conclusions drawn from the data. Indeed, the positive correlation between AN and PN (page 14, lines 334-336) indicates that the PN measure was for the attacker. That being the case, the only measure for playfulness is based on the attack component (initiating nape contact). This limits the overall conclusions that can be drawn, as rats may differ in likelihood of defending against such contact and in the types of defense used when doing so.

 We have rephrased the definition of PN behaviour to clarify that our observations focused on the active component of play (Lines 231-234). We have also stated the possible limitations of our approach in the Discussion and provided relevant references, however we also referred to previous research showing that PN and Being Pinned (complete rotation) are positively correlated in Lister Hooded rats (Lines 585-593). Assuming a correlation between the main attack component (PN, positively correlated with AN) and the main defence component (Being Pinned), we expected that PN would represent playfulness personality relatively well in this strain.


Page 10, “social preference test”: The failure to reveal a significant preference may be more a reflection of the test paradigm used than to a lack of preference. At this age, rats are equally likely to play with both familiar and unfamiliar partners and so, when confronted with rats to remain in close proximity, the cues may not be sufficient for the rat to discriminate. A conditioned place preference test, in which the I rat has a choice between a chamber that involves prior play with an H partner or L partner versus a chamber that involves prior play with an I partner, may be a more powerful tool to determine whether I rats have a preference.

After having tried this social preference test in our study, we fully agree with Reviewer 3´s criticism and we have added it in full in the Discussion at Lines 599-606, thank you.

Page 12, lines 279-282: What about AN?

We clarified that “The dependent variable AN was not analysed as it positively correlated with PN (see Results)” at Lines 332-333.
