# Supplementary material for: Play like me: Similarity in playfulness promotes social play
Source: PLoS One. 2019 Oct 24;14(10):e0224282. doi: 10.1371/journal.pone.0224282 (PMC6812795; doi:10.1371/journal.pone.0224282)
Supplement: Attachment — Submitted filename: Lampe et al - Response to Reviewers.docx [file pone.0224282.s006.docx]

**Response to Reviewers**

**Reviewer #1:**

The present version of the paper is much improved with the core findings now being more readily accessible. The core finding of importance is that heterogeneous groups of rats play less than do homogeneous ones. The unexpected finding was a failure to produce a consistent contagion effect. These major findings are now thoroughly discussed and their importance made clear. Less convincing is the ability to select for consistently high playing animals and how best to characterize behavioral differences across individuals and groups. I highlight these methodological issues below. In part, the problem is well beyond the confines of the present paper. Social play is, by its very nature, a social behavior, so isolating the contribution of one individual from that of the dynamics created by interacting with another animal is extremely hard. The approach used in the present paper is only partially successful, but the limitations that emerge are in themselves instructive. Hopefully, this paper will inspire others to explore different ways of identifying individuals with a different propensity to play in a way that can be dissociated from the influence of the partner. For example, repeatedly testing a subject with a standardized partner, such would be produced by injecting the partner with scopolamine. I have no further substantive concerns, but I do note some items that could be considered further in the Discussion to increase the impact that this paper. Certainly, some of these concerns should be considered in future studies.

Thank you for the overall positive feedback about our changes and for the constructive criticism on the methodology, please find our answers below.


Lines 145-146: Not clear why the ‘one or more’ is important for the prediction that “…(iv) one or more homogenous groups would play more than the heterogeneous groups…” Why not homogeneous groups should play more than heterogeneous groups? Clarify.

Our phrasing of this hypothesis was based on the fact that we didn´t know whether the average playfulness level of the group or the similarity in playfulness within the group was more important for play development. If the former was more important, then e.g. LLL groups should have played less than the heterogeneous groups, while if the latter was more important, then any homogenous group would have played more than any heterogeneous group. We have made a change to this hypothesis to make it more specific to our expectation: “(iv) homogenous groups (at least those with high or intermediate playfulness) would play more than the heterogeneous groups, as cage mates with similar motivation to play have been found to avoid each other less” (Lines 156-158).


Lines 183-186: The limitation of the method used is that, as the original grouping of rats is random, the multiple pair tests can only assess the relative playfulness of the individuals chosen to play together. As noted in the Introduction, there is evidence that the playfulness of the partner can increase or decrease the level of play expressed by an individual in a pair. While this procedure can attenuate the influence of the partners in assessing an individual’s playfulness (as described in lines 203-210), it does not eliminate that influence. Therefore, the labeling of rats as high or low players is relative, so that at a population level, it is not necessarily the case that that an H rat derived from a pair test would be on the extreme of the distribution representing H players. That these designations may not be robust is shown by the subsequent findings. The highest play frequency recorded is for the LLL group, especially on day 1 (Figure 3). Taking into account the variance, the statistical analyses show that there is no difference between the homogeneous groups (lines 414-416). This is confusing, because if the groups were formed by selecting the highest players and the lowest players, then how come the groups made up of high players are not playing more than the group constituted of low players? If anything, the low playing group is tending to play more (Figure 3). In this context, it is hard to understand what it means to be selected as a high or low player from the pair tests. As the consistency in a playful personality is central to the present paper, these discrepancies need to be adequately addressed. While these unexpected findings are discussed in terms of the effects of social manipulations on personality development (lines 573-577), they also need to be considered with regard to methodology. Perhaps the approach used to select more and less playful rats is not as robust as hoped. Although the lack of consistency between the pair-test and home cage levels of play is noted (lines 626-633), the broader issue of how best to identify high and low players selectively in a robust manner has not been resolved.

Reviewer 1 is correct in stating that our Play in Pairs test can only attenuate and not eliminate the effect of the play partners. We have now further highlighted this limitation in the Discussion (Lines 597-602) and suggested an improvement to the PIP test: “However, the fact that the playfulness scores in our study derived from only three play encounters between the subject rat and its three cage mates attenuates but does not exclude the possibility that such scores were influenced by the specific playfulness of the cage mates. One way to overcome this problem would be to refine the Play-in-Pairs test (e.g., by increasing the number of random, coetaneous partners used in the play encounters) to further improve the assessment of an individual´s playfulness”. However, we think that our result that LLL groups ended up playing the same or even more than III and HHH groups may have also occurred with a very robust assessment of playfulness personality. As also Reviewer 1 stated above, “isolating the contribution of one individual from that of the dynamics created by interacting with another animal is extremely hard”: if we paired our subject rat with e.g. a scopolamine treated play partner we would, yes, obtain a measure which is independent from the partner´s playfulness, but this measure may also not be representative of the actual play levels that the subject rat would express in a real life situation (i.e. different play experiences with different same age partners). In order to rank individuals within a population, we think that the playfulness of the subject rat should be measured by exposing this animal to many partners with varying levels of playfulness to obtain a reliable playfulness score for that individual. Assuming that the resulting playfulness scores would be more robust at the population level than the ones we measured in our study (i.e., many random play partners instead of only three) we could still observe the social manipulation effect on playfulness development that we discussed at Lines 591-597, because this effect would occur only in the social context where similarly low playful individuals (i.e., individuals that in average play little when paired with partners varying in playfulness) are housed together.


Lines 229-233: As defined, AN can underestimate the number of attacks launched, and PN can confound the role of the attacker with that of the attacker. Rats can begin to defend against a nape attack before contact with the nape is made, and by the definition given, these would not be counted as AN. Most pins arise when the recipient of an attack rotates to supine to protect its nape from being contacted (see Pellis & Pellis, 1987 cited), therefore pinning is not a property of the attacker alone, but the combination of its attack with the defensive maneuver used by the defender. For the latter, the use of the “Play Role Index” as a measure of relative asymmetry (lines 234-238) partially overcomes this problem as the rotated state of both pair mates is taken into account. Similarly, based on our recent experience with Lister hooded rats, it appears that as for Long Evans hooded rats, the correlation between attack and the specific attack outcome as defined by AN is quite tight, as is the correlation between overall defense and rotating to supine. However, in other strains, both AN and PN are not so tightly correlated with attack and defense (see Himmler et al., 2016 cited). For laboratories using other strains of laboratory rats, the methods used in the present paper with Lister hooded rats may not be as capable of detecting the effects of individual differences in playfulness. It would be worth noting these limitations in the Discussion to avoid the generalizability of the present findings from being compromised.

We have now noted these limitations at Lines 619-629 by stating that “Other aspects to be considered are that AN, as defined in this study, can underestimate the number of attacks launched, since rats can begin to defend against a nape attack before contact with the nape is made (Pellis et al., 1997). Also, the adoption by the play partner of defensive strategies other than complete rotation, such as partial rotation and evasion, may affect the quality of a play encounter. However, the initiations of social play in adolescent Lister hooded rats were shown to almost exclusively lead to pinning behaviour, with very few instances of other playful defensive strategies (Melotti et al., 2014; Lampe et al., 2017). Taken together, the main outcome measures in our study seem to represent playfulness personality well, yet it has to be taken into account that in other strains both AN and PN may not be tightly correlated with overall attack and defence behaviours (Himmler et al., 2016)”.

Lines 379-381: It is to be expected that AN and PN are correlated, as the supine position is most often achieved when the recipient of a nape attack rotates to supine to protect its nape from being contacted. However, the finding that PN is more frequent than AN is misleading. It is extremely rare that a rat rotates to supine in the absence of its partner launching an attack. Therefore, the discrepancy must arise because of the restricted measure of attack as defined by AN. As noted above, defense can be initiated before the attacker makes contact (see Himmler, B. T., Stryjek, R., Modlińska, K., Derksen, S. M., Pisula, W., & Pellis, S. M. (2013). How domestication modulates play behavior: A comparative analysis between wild rats and a laboratory strain of Rattus norvegicus. Journal of Comparative Psychology, 127, 453-464; Himmler, S. M., Modlińska, K., Stryjek, R., Himmler, B. T., Pisula, W., & Pellis, S. M. (2014). Domestication and diversification: A comparative analysis of the play fighting of the Brown Norway, Sprague-Dawley, and Wistar strains of laboratory rats. Journal of Comparative Psychology, 128, 318-327), which can lead to a rat beginning to rotate to supine before the AN configuration is achieved. Again, depending on the strain of rat used, this discrepancy can make a difference in how accurate either AN or PN is as a measure of attack (see Himmler et al., 2016 cited).

Based on this comment we have realised that an important part of the definition of PN was not clearly explained, so we have rephrased at Lines 245-246 that “every instance of the attacker raising its paws and pouncing again onto the already rotated rat was counted as a new event”. This last part of the PN definition alone explains why our PN measure was more frequent than AN. By counting also the repeated pouncing events while the play partner was already in supine position, our intention was to get a better measure of how motivated (or positively aroused) was the attacker. We added this explanation at Lines 246-247. However, since AN and PN were highly correlated in our study, we think that this difference in the definition did not have any major impact on our playfulness outcome measure.

Lines 585-593: The issue of play quality may also be important in interpreting the main effect shown in this study – that heterogeneous groups play less than homogeneous groups. For example, rats with certain types of brain damage initiate just as many attacks and are as likely to defend themselves as their intact controls, but the intact rats tend to avoid initiating play with such rats. Clearly, the brain-damaged rats are not playing in a manner found to be appropriate by the partner and so they become less attractive play mates (Pellis, S. M., Hastings, E., Shimizu, T., Kamitakahara, H., Komorowska, J., Forgie M. L., & Kolb, B. (2006). The effects of orbital frontal cortex damage on the modulation of defensive responses by rats in playful and non-playful social contexts. Behavioral Neuroscience, 120, 72-84). What such findings show is that compatibility in the amount of play initiated is only one factor of importance, the other is how the partner plays. While the present study makes a convincing case that the amount of play initiated by different rats is being reasonably well measured, it does not capture possible differences in the style of play. For the heterogeneous groups, discordance in how the players interact may be more important than in differences in how much play they initiate. This may be a useful avenue to explore in future studies.

Thank you for this useful and interesting suggestion, which we have now added to the Discussion at Lines 603-610 by stating that “Further research should focus not only on changes in play quantity, but also in play quality, derived by resorting of rats based on playfulness. For example, rats with certain types of brain damage initiate just as many attacks and are as likely to defend themselves as their intact controls, but the intact rats tend to avoid initiating play with such rats. This indicates that the brain-damaged rats are not playing in a manner found to be appropriate by the partner and thus they become less attractive play mates (Pellis et al., 2006). Similarly, discordance in how the players may have interacted in the heterogeneous groups could be at the basis of the observed differences in play amount between homogenous and heterogeneous groups”.

Line 725: “J Comp Psychol” should be “Int J Comp Psychol”

Changed, thank you.

**Reviewer #2:**

I think that the authors have made a good work with the revision of the MS by following all my suggestions. However, I still have concerns about the definition of some theoretical concepts. Before completely accepting the MS, I would like the authors address the following doubts.

Thank you for the overall positive feedback about our changes and for the additional suggestions provided.


Line 69: not only continuous interaction but also (and mainly) tight physical contact

We have made this addition at Line 69.


Lines 71-77: there is still confusion between the concepts of emotional contagion, Rapid Mimicry (RM) and behavioural synchronization. Emotional contagion occurs when a subject not merely perceives but mainly shares the same affective state of another individual. Certain forms of emotional contagion, but not all (e.g. consolation), can be mediated by mimicry, one of the phenomena at the basis of the behavioural synchronization. Since the mimic response is extremely rapid (< 1 sec), it occurs outside the conscious awareness and voluntary control. RM involve the Mirror Neuron System (MNS). Basing on the Perception-Action Model (Preston & de Waal, 2002), the involvement of MNS during the perception of an action or a facial expression activates shared representations: through the RM of an observed behaviour, emotional state related to that behaviour may be arisen in the observer.

We have rephrased and slightly expanded the explanation of the above concepts based on Reviewer 2´s suggestions (Lines 71-81), we think the link between them is now clearer.


Lines 78-83: the relation between emotional contagion and animal welfare is not still completely clear. Some studies have evidenced that the frequency of RM increases with the level of familiarity between subjects. For example, humans are likely to mimic more frequently the facial expressions of in-group- than out-group members (Seyfarth & Cheney, 2013). Moreover, within group RM is affected by the relationship quality of the interacting subjects (in humans, friends mimic each other’s smiles more than non-friends, Fisher et al., 2012). In this light, the replication and the sharing of fellows ' emotions promotes the development of tighter social relationships that contributes to social cohesion, one of the factors promoting animal welfare.

We are grateful for this suggestion and we have added it almost in full at Lines 91-98. Now the argument for a close link between emotional contagion and welfare is more robust: on a broader level, the first part explains that impaired (or improved) welfare of just one or few individuals can be transferred across (and thus affect) group members via emotional contagion; the second part focuses more specifically on the effect of familiarity and similarity between individuals on rapid mimicry.


Lines 508-509: I agree with the statement that different factors can affect play development, but the absence of evidence is not the evidence of absence. The fact that the presence of H and L seeders in the heterogeneous groups did not affect the play levels of I cage mates does not indicate the absence of emotional contagion during playful interactions. The authors investigated only PN and AN but they did not take into account other play patterns and play signals that can suggest the occurrence of the emotional contagion. I think that there is a still confusion between the concept of emotional contagion and playfulness personality.

From Reviewer 2´s comment we realised that a few sentences in the Discussion weren´t clearly expressed; in this study it wasn´t our aim to measure how exactly emotional contagion occurred during playful interactions, instead we wanted to measure whether emotional contagion (which likely occurs during play bouts) had a long-lasting impact on group-play development (and thus, likely, on welfare). So we have made some rephrasing to clarify this point. We now state that “This indicates that emotional contagion between seeder and cage mate rats, if it occurred, did not affect welfare in terms of expressed group play levels; it rather suggests that more complex group dynamics affected play development” (Lines 522-525) and “If emotional contagion between the seeders and their cage mates had affected group play development at any point during adolescence / early adulthood, the group with the highly playful seeder (HII) would have played more than the group with the low playful seeder (LII), however this was not the case. The absence of a long-lasting effect of emotional contagion on group play was supported by the results from the social preference test…” (Lines 631-635).
